# Contributions of Glycolipid Biosurfactants and Glycolipid-Modified Materials to Antimicrobial Strategy: A Review

**DOI:** 10.3390/pharmaceutics13020227

**Published:** 2021-02-06

**Authors:** Qin Shu, Hanghang Lou, Tianyu Wei, Xiayu Liu, Qihe Chen

**Affiliations:** Department of Food Science and Nutrition, Zhejiang University, Hangzhou 310058, China; 21713041@zju.edu.cn (Q.S.); louhanghang@zju.edu.cn (H.L.); 21913067@zju.edu.cn (T.W.); xiayuliu@zju.edu.cn (X.L.)

**Keywords:** glycolipids, nanocomposites, liposomes, antibacterial mechanism, anti-biofilm

## Abstract

Glycolipid biosurfactants are natural amphiphiles and have gained particular interest recently in their biodegradability, diversity, and bioactivity. Microbial infection has caused severe morbidity and mortality and threatened public health security worldwide. Glycolipids have played an important role in combating many diseases as therapeutic agents depending on the self-assembly property, the anticancer and anti-inflammatory properties, and the antimicrobial properties, including antibacterial, antifungal, and antiviral effects. Besides, their role has been highlighted as scavengers in impeding the biofilm formation and rupturing mature biofilm, indicating their utility as suitable anti-adhesive coating agents for medical insertional materials leading to a reduction in vast hospital infections. Notably, glycolipids have been widely applied to the synthesis of novel antimicrobial materials due to their excellent amphipathicity, such as nanoparticles and liposomes. Accordingly, this review will provide various antimicrobial applications of glycolipids as functional ingredients in medical therapy.

## 1. Introduction

**Figure 1:**
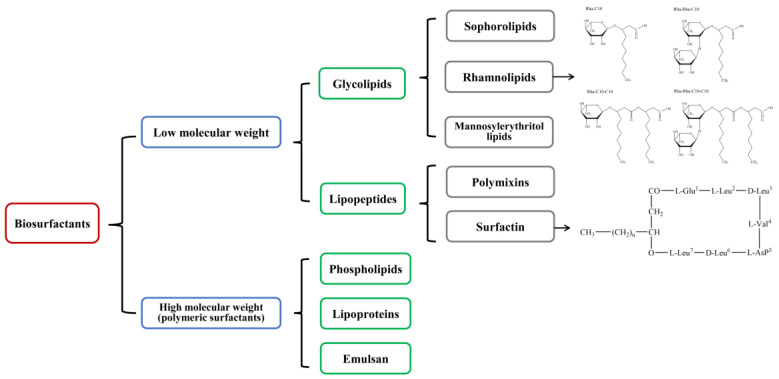
Main groups of biosurfactants.

Surfactants are a kind of amphiphilic molecules with hydrophilic moieties and hydrophobic moieties and perform excellent interfacial activity between gas, liquid, and solid surfaces. Since their broad range of applications has been put into the food industry, cosmetics, petroleum industry, and sewage treatment, surfactants have become one of the most widely used chemicals in industrial production. However, most chemically-synthesized surfactants derived from petroleum products, and the massive consumption of petroleum have caused resource exhaustion and environmental pollution. Thus, the exploitation of eco-friendly, biodegradable, and renewable alternative to surfactants has to be settled urgently. In recent years, the emerging of biosurfactants has gained more and more attention, and plenty of expectations have been potentiated due to their versatile functions, such as reducing surface tension, emulsifying activity, and biological properties [1]. Compared to synthetic surfactants, biosurfactants are more popular owing to their higher biodegradability, lower toxicity, thermostability, and tolerance in extreme conditions [2]. The production of biosurfactants is dependent on microbial fermentation of bacteria, fungi, and yeast strains based on low-cost carbon and nitrogen source, even industrial waste and oily byproducts, and the promising and sustainable strategy realizes low energy consumption in a large scale of production. According to their molecular weight, biosurfactants can be segmented into two groups: low molecular weight (LMW) and high molecular weight (HMW), as seen in Figure 1 [3]. Glycolipids [4] and lipopeptides [5] are representative biosurfactants with low molecular weight, such as rhamnolipids [6] and surfactin [7], and the high molecular weight group contains phospholipids [8], lipoprotein [9], and emulsan [8] et al.

Glycolipids are one of the most popular biosurfactants with low molecular weight and have been investigated thoroughly and intensively for biotechnological applications. As the name implies, glycolipids comprise two parts: carbohydrate moiety and fatty acid chains linked by a glycosidic bond, which acts as a hydrophilic role and hydrophobic role, respectively. The difference of carbohydrate moiety and fatty acid chains cause the diversity of glycolipids, and the sub-class normally contains rhamnolipids, sophorolipids, mannosylerythritol lipids, cellobiose lipids, trehalolipids, xylolipids, and so on [10]. Except for fundamental characteristics of biosurfactants, most glycolipid-producers are microorganisms isolated from oil-contaminated samples, which implied their potential applications in bioremediation as well as their production on renewable sources. Multiple biological properties have endowed glycolipids with wide potentiality in various fields. According to the literature, glycolipids have been confirmed to show potential anticancer effects and are expected to be efficient candidates for anticancer drugs [5]. For instance, rhamnolipids have shown a significant anticancer effect on human breast cancer cells MCF-7 in a dose-dependent manner [11]. Besides, glycolipids exert anti-inflammatory effects on human immune-related diseases as promising immunomodulators. Sophorolipids could modulate the immune response to decrease sepsis-related mortality in animal models, the underlying mechanism involves the reduction of nitric oxide and the regulation of inflammatory cytokines [12]. Remarkably, massive literature reveals that all kind of glycolipids possesses excellent antimicrobial activity against bacteria, fungi, and virus, and the physiological changes induced by glycolipids have been deeply investigated aiming at both planktonic and biofilm state of microorganism [4,6,13,14,15].

In the previous review [16], abundant work has been devoted to concluding the biotechnological production, functional properties, and potential applications of glycolipid biosurfactants. In this review, a comprehensive intensive study will be carried out directing the contributions of glycolipids and glycolipid-modified materials to antimicrobial therapy.

## 2. Glycolipids

### 2.1. Rhamnolipids 

**Figure 2:**
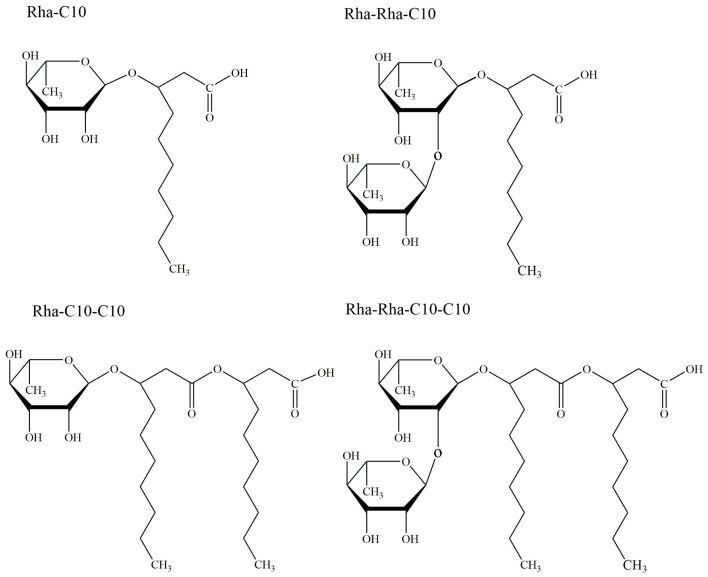
Common structures of rhamnolipids produced by *Pseudomonas* species.

Rhamnolipids (RLs) are one of the glycolipid-type biosurfactants, which are produced mainly by *Pseudomonas aeruginosa* and most frequently studied due to their effective surface activity and high yields of production [17]. It was firstly reported in 1946 that Bergström et al. discovered an oily glycolipid, named pyolipic acid, was produced by *Pseudomonas pyocyanea* (*P. aeruginosa*) after growing on glucose [18,19]. Soon after that, Jarvis and Johnson in 1949 have further confirmed the RL isolated from *P. aeruginosa* contained two β-hydroxydecanoic acids and two rhamnose moieties, which were linked through a glycosidic bond [20]. Then Edwards and Hayashi have verified the linkage between the two rhamnose moieties is an α-1,2-glycosidic linkage in 1965 [21]. Since then, extensive researches have been conducted on RL including broad aspects. So far, *P. aeruginosa* has been thoroughly investigated as a primary source of RLs with titers over 100 g/L [22], and Pseudomonas species are regarded as the main RL producers. Nevertheless, it has been reported that many other non-*Pseudomonas* isolates can produce RLs as well, leading to the structural diversity of RLs. For instance, there are some studies focused on the production of rhamnolipids by Burkholderia species, which have been shown to produce rhamnolipids that have longer alkyl chains than *P. aeruginosa*, such as *Burkholderia thailandensis* [23], *Burkholderia plantarii* [24], and *Burkholderia pseudomallei* [25]. Although the diversity of RL-producers has caused variations in the chemical structures of RL, the basic structure was composed of rhamnose moiety and lipid moiety, and the number of rhamnose and the length and number of carbon chain lead to the diversity of RL structure. Generally, there are mainly four types of RL structures produced by Pseudomonas species, including mono-rhamno-mono-lipid (Rha-C10), di-rhamno-mono-lipid (Rha-Rha-C10), mono-rhamno-di-lipid (Rha-C10-C10), and di-rhamno-di-lipid (Rha-Rha-C10-C10), as shown in Figure 2.

Earlier in 1971, the antimicrobial spectrum of rhamnolipids has been studied, suggesting the broad-spectrum antimicrobial activity functioned against common microbes including gram-positive and gram-negative bacteria, such as *Streptococcus faecalis*, *Staphylococcus aureus*, *Bacillus subtilis*, and *Proteus vulgaris* [26]. Whereafter, a large number of intensive and deep researches has been conducted about the microorganism sterilizing actions of rhamnolipids involving planktonic and biofilm cells, as seen in Table 1. As the most important pathogens, *Staphylococcus aureus* and *Staphylococcus epidermidis* were reported to be influenced as rhamnolipids have suppressed the growth of planktonic cells at MIC of 0.06 and 0.12 mg/mL, and dispersed pre-formed biofilms up to 93% [27]. Ferreira et al. provided evidence that gram-positive pathogens *Listeria monocytogenes*, *Bacillus cereus*, and *S. aureus* were more sensitive to rhamnolipids, indicating the sterilizing effects of rhamnolipids were pH-dependent [28]. Summarily, the inducement of bacterial death is attributed to cell lysis along with consequent leakage of cellular components, which was already confirmed by visualized proofs such as SEM and TEM pictures. As the target of rhamnolipids to planktonic bacteria, the cell membrane has been altered and damaged with an increase in cell permeability and reduction on cell surface hydrophobicity [28], because the unique amphiphilic character allows the interaction between rhamnolipids and phospholipids [29].

Interestingly, they found that gram-negative *Salmonella enterica* and *E. coli* showed resistance to rhamnolipids at all tested concentrations and all pH levels. Generally speaking, glycolipids have stronger antibacterial effects on gram-positive bacteria than gram-negative bacteria, which may be owing to the difference in cell membrane composition. The cell envelope of gram-negative bacteria consists of the outer membrane (lipopolysaccharides and phospholipids), peptidoglycan, and internal plasma membrane, which is more complex and defensive than gram-positive bacteria. The special structure made it difficult for glycolipids to enter gram-negative bacteria, while there is a common agreement that the underlying mechanism of antibacterial activity involves reducing membrane permeability, loss of intracellular constituents, and cell apoptosis induced by membrane lysis.

Generally speaking, the inducement of bacterial death is attributed to cell lysis along with consequent leakage of cellular components, which was already confirmed by visualized proofs such as SEM and TEM pictures. As the target of rhamnolipids to planktonic bacteria, the cell membrane has been altered and damaged with an increase in cell permeability and reduction on cell surface hydrophobicity [28], because the unique amphiphilic character allows the interaction between rhamnolipids and phospholipids [29]. 

Biofilm is considered as the sessile community of planktonic microorganism fixed at the solid surface, and plenty of researches have exhibited the anti-biofilm properties of rhamnolipids. Biofilms are an important concern among food processing industries, resulting in contamination of pipelines, corrosion of equipment, and final food spoilage. Araujo et al. discovered the biofilm of *P. fluorescens* and *L. monocytogenes* (biofilm-forming bacteria related to foodborne disease) was reduced up to 79% and 74% by purified rhamnolipids, respectively, while microbial adhesion was entirely inhibited in the culture medium [30]. Besides, oral health and hygiene have always been threatened by relating oral bacteria, and rhamnolipids were studied to eradicate the bacterial biofilm of oral disease. As one of the major etiological agents in dental caries, the adhesion of *Streptococcus mutans* on polystyrene surfaces was reduced by rhamnolipids, and its pre-formed biofilm was also disrupted according to Abdollahi et al. [31]. Similarly, Elshikh et al. also found that rhamnolipids from non-pathogenic *Burkholderia thailandensis* E264 revealed potent abilities to destruct mature biofilm of some oral pathogens (*Streptococcus oralis*, *Actinomyces naeslundii*, *Neisseria mucosa*, and *Streptococcus sanguinis*), forecasting their prospective oral-related applications against oral-bacteria biofilms [32]. In conclusion, the biofilm-inhibiting behavior of rhamnolipids was due to blocking the initial adhesion stage. To figure out the specific role of rhamnolipids playing in the biofilm formation process, Kim et al. investigated the physicochemical interactions between rhamnolipids and *Pseudomonas aeruginosa* biofilm layers [33]. They demonstrated that a decrease in surface free energy on the membrane, interaction with some EPS proteins, and loss of EPS amount were several key factors in rhamnolipid-mediated biofilm reduction.

Furthermore, rhamnolipids also perform excellent inhibiting effects on fungi including not only hyphal growth but also spore germination. For detrimental fungi in the plant, Kim et al. reported that rhamnolipids showed significant antifungal activity against *Phytophthora capsici* mainly due to a lytic effect on zoospores at a concentration of 10 mg/mL, and also suppressed the germination of zoospores and the growth of hypha [34]. Goswami et al. also confirmed the effectiveness of rhamnolipid in controlling *Colletotrichum falcatum* in vitro as well as in vivo. Results showed 100 mg/mL RL-DS9 exhibited 86.6% inhibition against *C. falcatum* spore germination, and in the same concentration, RL-R95 showed 83.3% inhibition. The antifungal mode came from disruption of the fungal membrane and made it possible for its application as an alternative fungicide to control red rot disease of sugarcane [35]. On the other hand, rhamnolipids are able to combat those harmful fungi derived from animals or humans. For instance, Sen et al. elucidated that purified rhamnolipid could effectively suppress spore germination and hyphal proliferation of *Trichophyton rubrum* in mice models at a concentration of 500 μg/mL, which can be a promising candidate to cure dermatophytic infections, known as the most prevalent superficial mycoses worldwide [36]. Meanwhile, the biofilm of fungi has always been detrimental to eliminate microbial contamination because of its high resistance and low living demands, yet rhamnolipids have outstanding performance in controlling fungi biofilm. Singh et al. discovered 90% of pre-formed *Candida albicans* biofilm on polystyrene surfaces was reduced by rhamnolipids in a dose-dependent manner [37]. Upon yeast type of fungi, the pre-formed biofilm of *Yarrowia lipolytica* was removed effectively by rhamnolipids at low concentrations, reported by Dusane et al. [38]. 

### 2.2. Sophorolipids

**Figure 3:**
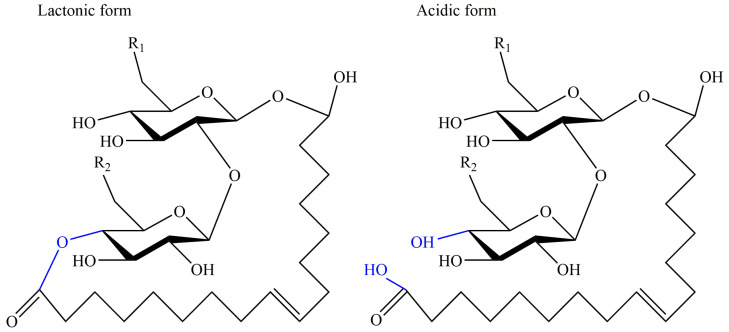
Common structures of sophorolipids: lactonic form and acidic form. R_1_ = OH or OCOCH_3_, R_2_ = OH or OCOCH_3._

Sophorolipids (SLs) are one of the representative glycolipid biosurfactants owing to their homogeneous product in high yield, which has been intensively investigated and commercialized by some companies [43]. The original discovery of sophorolipids was dated back to 1961 when Gorin et al. firstly revealed that an extracellular glycolipid mixture was produced by *Torulopsis magnoliae*, which was corrected as *Candida apicola* later in 1968 [44]. Simultaneously, Tulloch et al. also found extracellular glycolipids were yielded by *Candida bogoriensis* in 1968 [45]. In the next decades, sophorolipids have gained extensive interest and have been proved to be synthesized by multiple species of yeast strains like *Starmerella bombicola*, *Candida riodocensis*, *Candida stellate*, and *Wickerhamiella domercqiae*, growing on carbohydrates and lipophilic substrates with titers over 400 g/L [46,47,48]. Moreover, the diversity of producing species has determined the structure of their metabolic products. Normally, the molecular structure of sophorolipids is composed of a hydrophobic fatty acid tail of 16 or 18 carbon atoms and a hydrophilic carbohydrate head sophorose and can be divided into two main forms: acidic form and lactonic form, as shown in Figure 3. Specifically, a long chain of hydroxyl fatty acid was β-glycosidically attached to the sophorose moiety, and the carboxylic tail of the fatty acid is either free (acidic form) or esterified at the 6′- or 6′′-position (lactonic form). Furthermore, the variation of the structure is also reflected in the carbon number, unsaturation, and hydroxylation of the fatty acid chain in sophorolipids, depending on different kinds of carbon sources in microbial fermentation [49]. Thus, the structural difference has strongly influenced the biological and physicochemical activities and the degree of lactonization is the key factor. Generally, lactonized sophorolipid shows superior surface activity and antimicrobial effects and exhibits more application potential, which the acidic form demonstrates better forming capacity and solubility [50].

Sophorolipids have displayed diverse properties including emulsifier, lubricant, micelle formation, detergency, dispersibility, and wettability foaming, and their prominent antimicrobial activity has been deeply studied and applied in versatile field, seen in Table 2. Ankulkar et al. found semi-purified sophorolipids exhibited a different degree of antibacterial activity against pathogenic *Escherichia coli*, *Listeria monocytogenes*, and *Staphylococcus aureus* at minimum inhibitory concentrations (MIC) of 1000, 500, and 250 μg/mL, respectively [51]. Similarly, Fontoura et al. found gram-positive bacteria (*Enterococcus faecium*, *Staphylococcus aureus*, and *Streptococcus mutans*) were proved to appear more sensitive to sophorolipids than gram-negative bacteria (*Proteus mirabilis*, *Escherichia coli*, *Salmonella enterica subsp. enterica*), with difference in treating dose of 500 and 2000 μg/mL [52]. Nevertheless, some researches depicted the potent killing efficacy on both types of strains and revealed the otherwise working mechanism. For instance, Gaur et al. discovered that 60 mg/L sophorolipids isolated from *Candida glabrata* CBS138 killed 65.8% *Bacillus subtilis* and 4% *Escherichia coli*, and the further study confirmed the generation of reactive oxygen species (ROS) induced by sophorolipids has caused cell death [53]. 

For further practical application, sophorolipids were verified by Solaiman et al. to successfully inhibit the growth of five representative species of caries-causing oral bacteria: *Lactobacillus acidophilus*, *Lactobacillus fermentum*, *Streptococcus mutans*, *Streptococcus salivarius*, and *Streptococcus sobrinus*, suggesting the great potential of sophorolipids in oral health and hygiene [54]. Besides, sophorolipids have made contributions to reduce microbial contamination of *Clostridium perfringens* and *Campylobacter jejuni* in the poultry industry, which helps to lower enormous economic losses [55]. On the other hand, biofilm often fixes on the material surface such as medical devices, causes persistent microbial contamination and drug resistance, and has become prior trouble to be solved urgently. Recent research has revealed that sophorolipids have succeeded in scavenging biofilm of clinical strains (*Staphylococcus aureus*, *Pseudomonas aeruginosa*, and *Candida albicans*) on medical-grade silicone discs, resulting from their effective anti-adhesive ability [56]. Hence, there is immense potential to exploit sophorolipids as antimicrobial agents used in a variety of industries, such as poultry, food preservation, pharmaceutical industry, and medical apparatus and instruments. 

Aiming at pathogenic fungi, sophorolipids also have a significant influence on spore germination, mycelial growth, and biofilm formation. Firstly, sophorolipids show a broad antifungal spectrum involving *Colletotrichum gloeosporioides*, *Fusarium verticilliodes*, *Fusarium oxysporum* f. sp. *pisi*, *Corynespora cassiicola*, and *Trichophyton rubrum*, reported by Suparna et al. [57]. Haque et al. described the inhibition of sophorolipids on hyphal growth and biofilm formation of *Candida albicans*, and thought the downregulation of hypha specific genes was the reason for blocking biofilm formation [58]. Later, in further study, they elucidated that sophorolipids have increased the ROS production and expression of oxidative stress-related genes significantly in *Candida albicans*, ultimately leading to cell death by membrane perforation and necrosis [59]. To biological control of plant disease, Chen et al. exposed sophorolipids have a restraining effect on spore germination and hyphal tip growth of various plant pathogens, and the result showed pH solubility of sophorolipids had influenced their efficacy [58]. For zoonotic dermatophyte, sophorolipids also exerted an antifungal and anti-biofilm effect on *Trichophyton mentagrophytes* and are likely to treat cutaneous mycoses [60].

### 2.3. Mannosylerythritol Lipids

**Figure 4:**
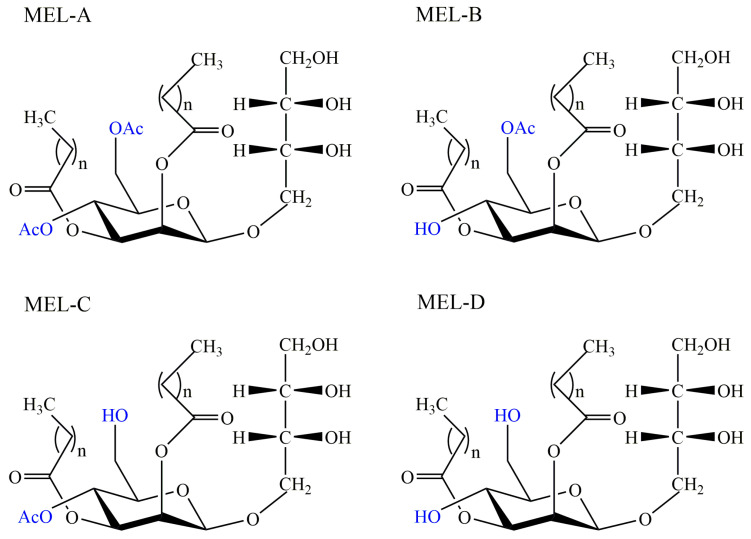
Different structures of mannosylerythritol lipids: Mannosylerythritol lipid (MEL)-A, MEL-B, MEL-C, and MEL-D, *n* = 2~18.

Mannosylerythritol lipids (MELs) are a type of glycolipid biosurfactants produced by *Pseudozyma* species mainly as well as *Ustilago* species [63]. Mannosylerythritol lipids were initially found and characterized in *Ustilago zeae* in the 1950s, but relevant researches were limited and unfocused in the following thirty years [64]. After the 1980s, a variety of strains were isolated as MEL-producers based on substrates of glucose and oil, such as *Pseudozyma antarctica*, *Pseudozyma tsukubaensis*, and *Pseudozyma hubeiensis* [65,66,67]. The basic molecular structure was composed of the mannose molecule linked to an erythritol residue at C-1′ and two fatty acids at the C-2′ and C-3′ [68]. Due to the different position and quantity of acetyl groups, these compounds can be divided into four groups: MEL-A (diacetylated at C-4′ and C-6′), MEL-B (monoacetylated at C-6′), MEL-C (monoacetylated at C-4′), and MEL-D (deacetylated) [69], shown in Figure 4. According to the literature, the structure of MEL depends on the carbon source primarily, and structural diversity confers to multiple interfacial and biological properties including emulsibility, non-toxicity, anti-cancer, and antioxidant activity, biodegradability, anti and environmental compatibility, showing their great application prospects in medical, environmental, cosmetic, pharmaceutical and food industries [70].

The bactericidal effects of MELs are of vital importance in their practical applications, as listed in Table 3. In 1993, Kitamoto et al. firstly pointed out that MELs showed significant antimicrobial activity, especially against Gram-positive bacteria [71]. Then, Fukuoka et al. also stated MEL-A and MEL-B have performed high inhibitory effects on *Micrococcus luteus*, and the reason was attributed to their solubilizing effect on bilayer biomembranes [72]. Similarly, the growth of gram-positive bacteria (*Bacillus megaterium* and *Bacillus subtilis*) was restrained by MEL-A and MEL-B reported by Recke et al., and they also found MELs showed moderate antifungal effects on *Candida magnoliae* [73]. Besides, Okuhira et al. compared the antimicrobial effects of MEL against two types of bacteria and summarized MEL selectively inhibited the proliferation of most gram-positive bacteria below the concentration of 50 μg/mL, but not gram-negative bacteria [74]. Furthermore, Nashida et al. synthesized 20 congeners of MELs through a chemical process and compared their inhibition on microbes, and the results illustrated not only the length of the alkyl chains but also the pattern of Ac groups on the mannose moiety were important factors for antibacterial activity [75]. 

So far, it has been reported that MELs have exhibited excellent sterilizing impact on foodborne bacteria and is expected to be a novel safe alternative to food preservative in food storage. Shu et al. investigated the antibacterial efficacy of MELs against *Bacillus cereus* and *Staphylococcus aureus* and concluded the mode of action involved the disruption of the cell membrane, leakage of cellular contents, the collapse of the whole cytoskeleton as well as induced cell apoptosis [76,77]. Liu et al. observed the same phenomenon in *Listeria monocytogenes* treated by MEL-A (32 μg/mL), and further transcriptome analysis demonstrated that the differentially expressed genes were enriched in the ABC transporter system, which verified the disorder of transmembrane protein played a key role in MEL-mediated cell death [78].

It is worth mentioning MELs are able to reduce the persistent contamination caused by fungi growth and microbial biofilm. For example, Yoshida et al. exposed the suppressive effects of MELs on the early infection behaviors of several phytopathogenic fungal conidia including *Blumeria graminis* f. sp. *tritici* (wheat powdery mildew fungi), *Colletotrichum dematium* (mulberry anthracnose fungi), *Glomerella cingulata* (strawberry anthracnose fungi), and *Magnaporthe grisea* (rice blast fungi), presumably owing to their inhibition to conidial germination, and anticipated the future application of MELs as novel agricultural chemical pesticides [79]. On the other hand, it is evident that MELs showed potent anti-biofilm activity against *Staphylococcus aureus* through attachment inhibition and biofilm dispersal, which was explained as the involvement of biosurfactants in microbial adhesion and desorption [77,80].

### 2.4. Cellobiose Lipids 

**Figure 5:**
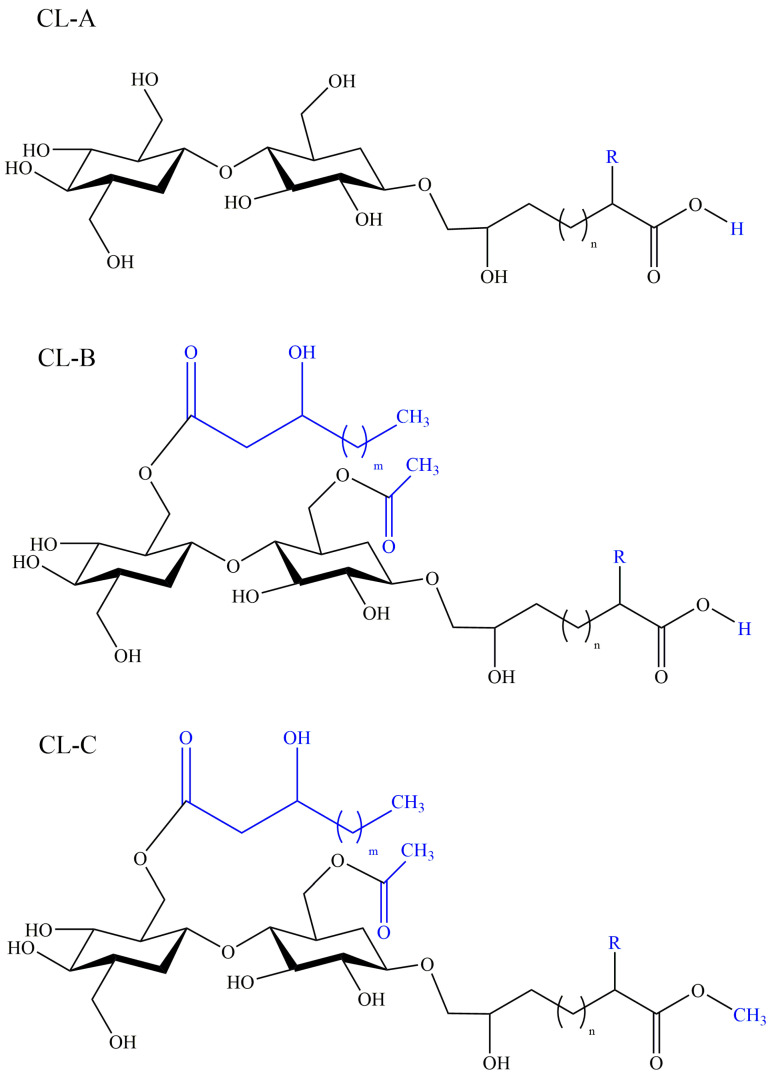
Structures of cellobiose lipids isolated from *Ustilago maydis*: CL-A, CL-B, and CL-C. R = H or OH, *m* = 2 or 4.

Cellobiose lipids (CLs) are a kind of glycolipid biosurfactants with less research than other glycolipids. In 1951, cellobiose lipids were firstly reported as ustilagic acid (UA) with antibiotic activity, produced by phytopathogenic fungi *Ustilago maydis* [81], and the structure was then characterized as three forms: CL A, CL B, and CL C, shown in Figure 5 [82]. Subsequent researches have revealed a diversity of CL-producers including *Cryptococcus huminola*, *Pseudozyma fusiformata* [83], *Pseudozyma graminicola* [84], *Pseudozyma. flocculosa* [85], and *Sporisorium scitamineum* [86] cultivated on glucose, vegetable oil, and alkane. The basic structure of cellobiose lipids consist of a cellobiose moiety as the hydrophilic group and a fatty acid chain as the hydrophobic group, and the variants can differ in the presence or absence of hydroxyl group (R = H or OH) or the length of fatty acid chain as well as an additional easter group (CL C) [82]. The studies suggest the structure of cellobiose lipids is determined by their producing microorganisms. Cellobiose lipids have the advantage of biodegradability, low toxicity, emulsibility, surface activity, a wide range of pH tolerance and thermostability over chemical surfactants, and have great potential employed in the food industry, medical field, and environmental protection.

The antifungal activity of cellobiose lipids has been investigated thoroughly since Haskins et al. firstly discovered the antibiotic activity of ustilagic acid (namely cellobiose lipids) in 1951 [81]. The targeting fungi contains yeast and filamentous fungi, such as *Saccharomyces cerevisiae* [87], *Cryptococcus*, and *Candida* species [83], and the underlying antifungal mechanism is always associated with the structural and functional disorder of cell membrane caused by cellobiose lipids. For instance, Puchkov et al. illuminated that the intercalation of cellobiose lipids into the liposomal lipid matrix resulted in the increasing permeabilization of cytoplasmic membrane, ATP leakage, and high susceptibility of targeted cells, and considered this antifungal mode of action was relevant to detergent-like properties [88]. This membrane-damaging activity of cellobiose lipids was also proven by Kulakovskaya et al. that CL has induced K^+^ leakage and inhibited polyphosphate accumulation in *Saccharomyces cerevisiae* cells [89]. Moreover, further study displayed the sensitivity of *Saccharomyces cerevisiae* cells to antifungal cellobiose lipids is up to culturation components, especially carbon source, and speculated that the long-chained polyP participated in the viability restoring of ethanol-grown cells after treated by cellobiose lipids [87]. Besides, flocculosin is known as one of the effective components in biological control agents of powdery mildew fungi and has been confirmed to show inhibitory effects on pathogenic fungi, and further work revealed that flocculosin caused direct damage to the membrane surface of sensitive micro-organisms, such as *Staphylococcus* species and *Candida albicans*, which implied the membrane-mediated antimicrobial mechanism was suitable for gram-positive bacteria [90,91]. Consequently, CLs are defined as a novel safe and natural fungicide more exactly and allowed to become membrane-penetrating agents against yeast and fungal cells.

## 3. Glycolipid-Modified Materials 

### 3.1. Nanocomposites

**Figure 6:**
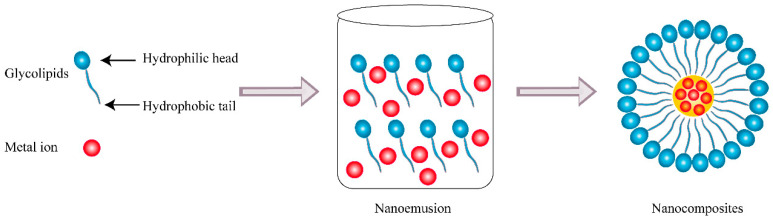
Schematic diagram of the synthetic pathway of nanocomposites based on glycolipids.

Nanotechnology is a new burgeoning technology dealing with nanoscale dimensions of particles that can produce novel multifunctional materials and devices with a variety of applications. The size of nanoparticles ranges from 1 to 100 nm and the combination of two or more compounds of materials, in which at least one compound has a dimension less than 100 nm in size, can be defined as nanocomposites [92]. Compared to normal materials, nanocomposites have the advantage of physicochemical and combined biological properties and have attracted interest in various technological and environmental areas such as medicine, energy, cosmetics, electronics, packaging, coatings, and biotechnology. As a carrier, nanocomposites are able to deliver substances such as drugs and achieve the effect of target therapy and release control, which have great potential in practical applications of antitumor therapy [93]. The synthesis of nanocomposites is usually based on chemical and physical methods, while the former is the most widely adopted and highly efficient for large-scale and high-performance production of nanocomposites. Nevertheless, the nanoparticles are often synthesized along with hazardous and toxic byproducts during the chemical process, which may pose a threat to public hygiene and environment. As a multifunctional agent, biosurfactants have gradually gained more attention in recent years and played a crucial role in the green synthesis of nanocomposites as seen in Figure 6, owing to their superior biocompatibility, biodegradability, high efficiency of stabilization, and dispersion. Firstly, biosurfactants could enhance the stability of nanocomposites by reducing the interfacial tension and facilitating nanoemulsion formation. Furthermore, the presence of biosurfactants prevents nanoparticles from aggregation and promotes dispersion in organic solvents or water as a capping agent. On the other hand, biosurfactants could act as a reducing agent in the synthesis of nanocomposites, which effectively simplifies the producing procedures with better control [94]. Except for their physical characteristics, biosurfactants will improve the biological properties of nanocomposites as a functional adjuvant involving antioxidant, antimicrobial, and anticancer effects.

In the past decade, the rapid development of nanotechnology has been emphasized and provided more opportunities in the antimicrobial field. Nanomaterials have been proved to be effective to inhibit cell growth of various microorganisms even multi-drug-resistant bacteria, and show less possibility to induce microbial resistance [95]. According to the literature, the antimicrobial modes could be summarized as increasing cell membrane permeability, inhibiting efflux pump, and generating reactive oxygen species (ROS) [96,97]. Therefore, glycolipids, as a typical kind of biosurfactants, have been employed in nanomaterial production due to their excellent emulsifying and antimicrobial properties, as listed in Table 4. 

Das et al. reported that rhamnolipids could be used to stabilize the synthesis of silver nanoparticles, and the antibacterial and antifungal activity of both silver nanoparticles and purified rhamnolipids against four strains of bacteria (*Staphylococcus aureus*, *Bacillus subtilis*, *Escherichia coli*, and *Klebsiella pneumoniae*) and two fungi (*Aspergillus niger* and *Aspergillus flavus*) was studied [98]. The comparative result suggested silver nanoparticles are more effective when inhibiting the microbial growth of all kinds than purified rhamnolipids. Joanna et al. also synthesized silver nanoparticles (AgNPs) through chemical and biological processes, respectively, and discovered the existence of rhamnolipid significantly increased the stability of biogenic AgNPs and enhanced their antimicrobial activities [99]. The rhamnolipid-involved biogenic AgNPs exhibited more effective inhibition on gram-positive bacteria and phytopathogenic fungi than gram-negative bacteria, probably owing to the difference in bacterial membrane structure. Furthermore, the interactions between biogenic AgNPs and DNA were investigated and the DNA particles were significantly observed to accumulate around the AgNPs densely, potentially due to the strong affinity between metal nanoparticles and nitrogen bases with the hydrogen bonds. On the other hand, rhamnolipids help to stabilize the meatal nano-carrier when encapsulating various substances, such as carvacrol [100]. The rhamnolipid-stabilized carvacrol-loaded zein nanoparticles have been verified to suppress the growth of phytopathogens *Pseudomonas syringae* and *Fusarium oxysporum*, which might result from the synergistic antimicrobial effects of carvacrol, zein, and rhamnolipids.

Interestingly, there is often a win-win situation that glycolipid-modified nanocomposites exert potent inactivating function against both planktonic cells and persistent biofilm. Marangon et al. elucidated that a combination of rhamnolipid and chitosan in nanoparticles boosts their antimicrobial efficacy on *Staphylococcus* strains, and the reason was attributed to the increased local delivery of chitosan and rhamnolipid at the cell surface and, consequently, to their targets in gram-positive bacteria [101]. Especially, although chitosan could not penetrate deeply in the biofilm through adsorption on the surface, the nanoparticles enabled the antibacterial rhamnolipid to release and fill the whole biofilm, rendering devastation of the biofilm structure and death of dormant cells. Another mechanism of biofilm eradication for nanomaterials is based on the anti-adhesive property of glycolipid biosurfactant, and it has been reported that rhamnolipid-coated silver and Fe_3_O_4_ nanocomposites exert more anti-biofilm efficacy against pathogenic *Pseudomonas aeruginosa* and *Staphylococcus aureus* than individual RL or bare nanoparticles [97].

Sophorolipids could also participate in the fabrication of nanomaterials and be considered as dual roles including a biostabilizer and a biofunctionalizing agent. For instance, zinc oxide nanoparticles (ZON) modified by sophorolipids have shown stronger inhibitory activity against *Salmonella enterica* and *Candida albicans* compared with naked ZON [96]. Generally, gram-positive bacteria reveal more sensitivity to sophorolipids than gram-negative bacteria, while sophorolipid capped gold nanoparticles (AuNPs-SL) exhibited antibacterial properties against both gram-positive and gram-negative bacteria via binding to cell membrane, disintegrating cell membrane, leaking intracellular constituents, and interfering the enzyme activity [102]. Aiming at biofilm elimination, the acidic sophorolipid was used to encapsulate hydrophobic curcumin in order to form nanostructures with better dispersibility in water [102]. The sophorolipid–curcumin nanocomposites have the capability to scavenge the biofilm of *Pseudomonas aeruginosa* as a quorum quencher. 

Although the researches of MELs involved in nanomaterial synthesis are not as many as others, their applications in nanotechnology are emerging and advancing latterly. Firstly, Wu et al. embedded chitosan nanoparticles into essential oils using MEL-A as an emulsifier and observed an apparent inhibition zone against *Staphylococcus aureus* around the chitosan-based nanoparticles [103]. Subsequently, Bakur et al. successfully fabricated metallic nanomaterials including silver, zinc oxide, and gold nanoparticles mediated by MEL-A, and all of them exert inhibitory efficacy against pathogenic gram-positive and gram-negative bacteria [104]. These findings indicated that MELs play a crucial role in the rapid biosynthesis of metallic nanoparticles and enhance the antimicrobial property, yet the underlying mechanism of inhibition still needs further study. 

### 3.2. Liposomes 

Liposomes are small spherical vesicles and normally possess a closed lipid bilayer resembling a cell membrane and an aqueous inner chamber, which could encapsulate hydrophilic and hydrophobic molecules, respectively. As multifunctional drug-carriers, liposomes have the advantages of biocompatibility, stability, membrane fusion, gene transfection, controlled release, and entrapment protection, widely applied in pharmaceutical, cosmetics, and food fields [107]. There is a diversity of liposome structures including single or multiple lipid bilayers, and one or more compartments. The variations of their properties are depending on the composition, size, surface charge, and producing method. The size of liposomes is ranging from nanometers to micrometers, and it will be called nanoliposome when the size is less than 100 nm. Since the first liposome Doxil^®^ was approved by FDA for cancer therapy in 1995, there are more and more FDA-approved liposome-based pharmaceuticals applied into clinical treatments in the following decades, involving anticancer, antimicrobial, and antiviral therapies [108]. For example, rifampicin-loaded liposomes were frequently reported [109]. For lung inhalation therapy, a polyelectrolyte complex based on chitosan and carrageenan was used to coat rifampicin-loaded vesicles and obtain a dry powder by spray-drying [110]. Furthermore, inhalable polymer-glycerosomes were proved to be safe and effective carriers for rifampicin delivery to the lungs, which enhanced the local pharmacological activity of rifampicin, reduced possible side effects, and improved drug efficacy [111].

Thereby, the broad encapsulating properties of liposomes come up with a novel strategy to inactivate microorganisms with reduced resistance. It is easy for natural or synthetic antimicrobial agents incorporated in liposomes to reach intracellular targets via the fusion of lipid bilayer and cell membrane, hence exerting high therapeutic efficacy. Therefore, glycolipids could be embedded into liposomes together with other bactericides, thereby realizing multiple antimicrobial effects on gram-positive, gram-negative, and fungi. Nevertheless, this research direction has been rarely investigated. 

Except for acting as bioactive substances, glycolipids are able to self-assemble as a closed lipid bilayer, and the formed spherical vesicles are orderly arranged through exposed hydrophilic glycosyls and tail-to-tail hydrophobic fatty acid chains. Interestingly, MELs specialize in self-assembly properties and form vesicle structure with thermodynamic stability, and have great potential to participate in liposome fabrication and stabilize the bilayer structure [112]. For example, Wu et al. demonstrated the chitosan-coated liposome with loading betulinic acid was modified by MEL-A, and the results implied MEL-A boosted the antioxidant effect of betulinic acid [113]. Fan et al. successfully prepared stable vesicles consisting of l-α-phosphatidylcholine (PC) and MEL-A by a thin lipid film hydration technique and evaluated their structural characterization, stability, and encapsulation efficiency [114]. The consequence showed the addition of MEL-A changed the dispersibility of PC in water, rendering the formation of vesicle solution with smaller size and higher encapsulation efficiency. Moreover, anthocyanins embedded in vesicles exhibited improved antioxidant activity and higher retention rate in simulated gastrointestinal fluid environment than bare anthocyanins, mainly owing to the protection of vesicles. In the antimicrobial field, sophorolipid has been reported to formulate noisome with amphotericin B, and the complex was proved to inhibit the growth of planktonic cells and destruct mature biofilm of *Candida albicans*, presumably through downregulating expressions of the genes responsible for hyphae formation [115]. These findings anticipate the prospective development of liposomes based on glycolipids.

## 4. Conclusions and Perspectives 

**Figure 7:**
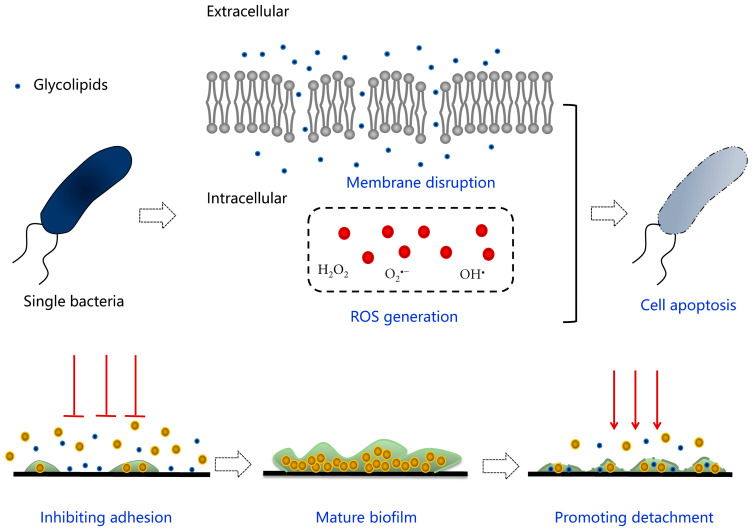
Schematic diagram of the antimicrobial mechanism of glycolipids involved in planktonic cells and biofilms.

Glycolipids, as a kind of representative biosurfactants, are famous for versatile bioactivities and have immerse potential to be put into practical applications due to their environmental friendliness compared to chemical surfactants. Deep insight into the predominant antimicrobial behaviors of glycolipids reveals several underlying mechanisms when targeting planktonic or biofilm state of microorganisms, as seen in Figure 7, which provides a theoretical basis for exploiting novel safe antimicrobial agents applied in food, cosmetics, and medical fields. The induced death of a single cell could be attributed to the following reasons. The membrane permeabilization is considered as the prior factor leading to cell lysis and apoptosis. Specifically, glycolipids are able to insert into the lipid bilayer relying on their unique similar structure, leading to the deformation and collapse of membrane skeleton physically as well as a conformational change of related proteins functionally. As the main member of membrane proteins, ATP-binding cassette (ABC) transporters are responsible for transmembrane migration of material and energy transformation via ATP hydrolysis, and it has been proven that glycolipids have rendered the disorder of movement across the membrane and material supplement by regulating the different expression of related genes, which might be internal reason accounting for the final cell apoptosis [78,116]. Except for membrane-mediated modes, correlational researches also discovered the generation of ROS in bacteria and fungi [53,59]. Generally, it is a common phenomenon that ROS is generated by normal cells during oxygen respiration and metabolism, while ROS production is increased due to redox-cycling agents, membrane disrupters, and antibiotics [117]. As a result, oxidative stress emerges when a cell is not able to detoxify the excessively accumulated ROS, leading to cell death by necrosis or apoptosis. On the other hand, eliminating refractory microbial contamination caused by persistent and resistant biofilm has become a huge challenge to be solved urgently. Glycolipid-involved strategy to tackle microbial biofilm is mainly aiming at blocking the adhesive stage and promoting the dispersal stage during the biofilm formation [118]. The anti-adhesive behavior can be attributed to the intrinsic antimicrobial and physiochemical characteristics of glycolipids, causing reduction of the cell density and changes in hydrophilic/hydrophobic characteristics of treated surfaces. The promotion of biofilm dispersal is related to the permeation of glycolipids into the biofilm matrix, resulting in detachment of extracellular matrix (EPS) and disintegration of the whole biofilm community. Although glycolipids play extraordinary roles in antimicrobial work, they behave less friendly in the human immune system. The literature revealed rhamnolipids has functioned as immune modulators and virulence factors, leading to rapid necrotic killing of polymorphonuclear leukocytes [119], early infiltration of primary human airway epithelia [120], and killing the myofibroblasts [118].

Nano-scale approaches have been rapidly progressed and attract concentration globally on controlling microbial contamination in medical and healthcare applications. Nano-carriers (such as nanoparticles and liposomes) are regarded as crucial drug delivery systems due to their great dispersity, high encapsulation efficiency, and stability. Glycolipids have been reported to play an important role in the modification of these materials both structurally and functionally, relying on their special amphipathicity and self-assembling activity as well as various biological activities. Therefore, the antimicrobial effect of glycolipid-modified material has been augmented evidently because of the combination of effective compounds. The modification of new materials based on glycolipids prompts the exploitation of novel antimicrobial agents applicable to different requirements.

Furthermore, the synergic work of glycolipids and antibiotics or other antimicrobials on microorganisms is on trial and has made initial progress. Sophorolipids was confirmed to work co-jointly with tetracycline to cause swelling and morphological damage of methicillin-resistant *Staphylococcus aureus* [119]. The underlying mechanism was speculated that the self-assembled glycolipids could span through the structurally alike bacterial cell membrane and thereby facilitate the entry of antibiotics [120]. At present, antimicrobial resistance has posed a serious threat to public health and hygiene, leading to an increasing number of ineffective antibiotics. Nevertheless, developing new antibiotics is time- and money-consuming, and difficult, which has the risk of new drug-resistance emerging. Hence, the great potential of glycolipids to attenuate microbial resistance will be a hot spot and presents a new strategy to inactivate drug-resistant microorganisms via the synergy of antibiotics and glycolipids, which will make a break in the antimicrobial field. 

## Figures and Tables

**Table 1:** Antimicrobial effects of rhamnolipids reported to date.

Producing Microorganism	Target Microorganism	Microbial Type	Inhibitory Effects	Reference
*Pseudomonas aeruginosa* PA1	*Listeria monocytogenes*	Gram-positive	Anti-biofilm effects	[30]
	*Pseudomonas fluorescens*	Gram-negative		
*Pseudomonas aeruginosa* OBP1	*Staphylococcus aureus*	Gram-positive	Antibacterial effects	[27]
	*Klebsiella pneumoniae*	Gram-negative		
*P. aeruginosa* DS10-129	*Staphylococcus epidermidis*	Gram-positive	Anti-biofilm effects by disrupting the initial adhesion	[39]
	*Staphylococcus* *aureus*			
	*Streptococcus* *salivarius*			
	*Candida tropicalis*	Fungi		
*Pseudomonas* sp. PS-17	*Bacillus subtilis*	Gram-positive	Antibacterial effects	[40,41]
—	*Bacillus cereus*	Gram-positive	Antibacterial effect depending on pH	[28]
	*Escherichia coli*	Gram-negative		
	*Salmonella enterica*	Gram-negative		
*Pseudomonas aeruginosa* MN1	*Streptococcus mutans*	Gram-positive	Anti-biofilm effects	[31]
—	*Helicobacter pylori*	Gram-negative	Anti-biofilm effects	[42]
*Pseudomonas aeruginosa* strain B5	*Cercospora kikuchii*	Fungi	Inhibiting spore germination and hyphal growth	[34]
	*Phytophthora capsici*			
*—*	*Yarrowia lipolytica*	Fungi	Anti-biofilm effects	[38]
*Pseudomonas aeruginosa* DSVP20	*Candida albicans*	Fungi	Anti-biofilm effects	[37]
*Pseudomonas aeruginosa* DS9	*Colletotrichum falcatum*	Fungi	Inhibiting spore germination and mycelial growth	[35]
*Pseudomonas aeruginosa* SS14	*Trichophyton rubrum*	Fungi	Inhibiting spore germination and hyphal proliferation	[36]

**Table 2:** Antimicrobial effects of sophorolipids.

Producing Microorganism	Target Microorganism	Microbial Type	Inhibitory Effects	Reference
*Candida glabrata* CBS138	*Bacillus subtilis*	Gram-positive	Antibacterial effects	[53]
	*Escherichia coli*	Gram-negative		
*Candida* *tropicalis* RA1	*Staphylococcus aureus*	Gram-positive	Antibacterial effects	[51]
	*Listeria monocytogenes*			
	*Escherichia coli*	Gram-negative		
*Candida bombicola*	*Enterococcus faecium*	Gram-positive	Antibacterial effects	[52]
	*Staphylococcus aureus*			
	*Streptococcus mutans*			
	*Proteus mirabilis*,	Gram-negative		
	*Escherichia coli*			
	*Salmonella enterica subsp. enterica*			
*Candida bombicola* ATCC 22214	*Lactobacillus acidophilus*	Gram-positive	Antibacterial effects	[54]
	*Lactobacillus fermentum*			
	*Streptococcus mutans*			
	*Streptococcus salivarius*			
	*Streptococcus sobrinus*			
*Starmerella* (*Candida*) *bombicola*	*Clostridium perfringens*	Gram-positive	Antibacterial effects	[55]
	*Campylobacter jejuni*	Gram-negative		
*Candida bombicola*	*Escherichia coli*	Gram-negative	Antibacterial effects	[61]
*Candida bombicola* ATCC 22214	*Staphylococcus aureus*	Gram-positive	Anti-biofilm effects	[56]
	*Pseudomonas aeruginosa*	Gram-negative		
	*Candida albicans*	Fungi		
*Starmerella* (*Candida*) *bombicola* MTCC1910	*Candida albicans*	Fungi	Antifungal and anti-biofilm effects	[58,59]
*Rhodotorula babjevae* YS3	*Colletotrichum gloeosporioides*	Fungi	Antifungal effects	[57]
	*Fusarium verticilliodes*			
	*Fusarium oxysporum f.* sp. *pisi*			
	*Corynespora cassiicola*			
	*Trichophyton rubrum*			
*Wickerhamiella domercqiae*Y2A	*Phytophthora infestans*	Fungi	Inhibiting spore germination and mycelial growth	[60]
	*Fusarium* sp.			
	*Fusarium concentricum*			
	*Fusarium oxysporum*			
	*Pythium ultimum*			
	*Pyricularia oryzae*			
	*Rhizoctorzia solani*			
	*Alternaria kikuchiana*			
	*Gaeumannomyces graminis* var*. tritici*			
	*Phytophthora infestans*			
*Rhodotorula babjevae* YS3	*Trichophyton mentagrophytes*	Fungi	Antifungal and anti-biofilm effects	[62]

**Table 3:** Antimicrobial effects of mannosylerythritol lipids.

Producing Microorganism	Target Microorganism	Microbial Type	Inhibitory Effects	Reference
*Pseudozyma aphidis* T-34	*Micrococcus luteus*	Gram-positive	Antibacterial effects	[72]
*Pseudozyma aphidis* CBS 517.83	*Bacillus megaterium*	Gram-positive	Antibacterial effects	[73]
	*Bacillus subtilis*			
	*Staphylococcus aureus*			
	*Candida magnoliae*	Fungi		
*Pseudozyma aphidis*	*Bacillus cereus*	Gram-positive	Antibacterial effects	[76]
*Pseudozyma aphidis DSM 70725*	*Staphylococcus aureus*	Gram-positive	Antibacterial and anti-biofilm effects	[77]
*Pseudozyma aphidis* DSM 70,725	*Listeria monocytogenes*	Gram-positive	Antibacterial effects	[78]
	*Micrococcus luteus*	Gram-positive	Antibacterial effects	[75]
	*Staphylococcus aureus*			
	*Enterococci faecalis*			
	*Enterococci faecium*			
*Pseudozyma aphidis* NBRC 10182	*Streptococcus bovis*,	Gram-positive	Antibacterial effects	[74]
	*Lactobacillus ruminis*			
	*Eubacterium ruminantium*			
	*Butyrivibirio fibrisolvens*			
	*Ruminococcus albus*			
	*Ruminococcus flavefaciens*			
*Lactobacillus casei*	*Staphylococcus aureus*	Gram-positive	Anti-biofilm effects	[80]
*Pseudozyma* yeast	*Blumeria graminis f*. sp. *tritici*	Fungi	Inhibition of conidial germination.	[79]
	*Colletotrichum dematium*			
	*Glomerella cingulata*			
	*Magnaporthe grisea*			

**Table 4:** Antimicrobial effects of glycolipid-modified nanocomposites.

Name	Glycolipid Type	Antimicrobial Effects	Reference
Rhamnolipid-stabilized carvacrol-loaded zein nanoparticles	Rhamnolipid	Antibacterial and antifungal activity against *Pseudomonas syringae* and *Fusarium oxysporum.*	[100]
Silver nanoparticles	Rhamnolipid	Antibacterial and antifungal activity against *Staphylococcus aureus*, *Bacillus subtilis*, *Escherichia coli* and *Klebsiella pneumoniae*, *Aspergillus niger*, and *Aspergillus flavus.*	[98]
AgNPs	Rhamnolipid	Antibacterial activity against gram-positive bacteria and antifungal activity against phytopathogens.	[99]
Silver and iron oxide nanoparticles	Rhamnolipid	Antibacterial and anti-adhesive properties against biofilms formed by *Pseudomonas* aeruginosa and *Staphylococcus aureus*.	[97]
Chitosan/rhamnolipid nanoparticles	Rhamnolipid	Antibacterial and anti-biofilm activity against *Staphylococcus* strains.	[101]
ZnO Nanoparticle	Sophorolipid	Antibacterial activity against *Salmonella enterica* and *Candida albicans.*	[96]
Curcumin-sophorolipid nanostructures	Sophorolipid	Antibacterial activity anti-biofilm activity against *Pseudomonas aeruginosa.*	[105]
Gold Nanoparticles	Sophorolipid	Antibacterial and anti-biofilm effects on *Staphylococcus aureus* and *Vibrio cholerae*	[102]
Chitosan Nanoparticles	MEL-A	Antibacterial activity against *Staphylococcus aureus*	[103]
MEL@AgNPs, MEL@ZnONPs and Ag-ZnO/MEL/GA	MEL-A	Antibacterial activity against *Escherichia coli*, *Salmonella enterica*, *Bacillus cereus* and *Staphylococcus aureus.*	[106]
Gold nanoparticles (AuNPs)	MEL-A	Antibacterial activity against gram-positive and gram-negative bacteria.	[104]
